# New insights into healthy ageing, inflammageing and frailty using metabolomics

**DOI:** 10.3389/fragi.2024.1426436

**Published:** 2024-07-09

**Authors:** Genna Abdullah, Asangaedem Akpan, Marie M. Phelan, Helen L. Wright

**Affiliations:** ^1^ Institute of Life Course and Medical Sciences, University of Liverpool, Liverpool, United Kingdom; ^2^ Division of Internal Medicine, University of Western Australia, Bunbury, WA, Australia; ^3^ Faculty of Health Sciences, Curtis University, Bunbury, WA, Australia; ^4^ Department of Geriatric Medicine, Bunbury Regional Hospital, Bunbury, WA, Australia; ^5^ Institute of Systems, Molecular and Integrative Biology, University of Liverpool, Liverpool, United Kingdom; ^6^ High Field NMR Facility, Liverpool Shared Research Facilities University of Liverpool, Liverpool, United Kingdom

**Keywords:** healthy ageing, inflammageing, frailty, metabolism, metabolomics

## Abstract

Human ageing is a normal process and does not necessarily result in the development of frailty. A mix of genetic, environmental, dietary, and lifestyle factors can have an impact on ageing, and whether an individual develops frailty. Frailty is defined as the loss of physiological reserve both at the physical and cellular levels, where systemic processes such as oxidative stress and inflammation contribute to physical decline. The newest “omics” technology and systems biology discipline, metabolomics, enables thorough characterisation of small-molecule metabolites in biological systems at a particular time and condition. In a biological system, metabolites—cellular intermediate products of metabolic reactions—reflect the system’s final response to genomic, transcriptomic, proteomic, epigenetic, or environmental alterations. As a relatively newer technique to characterise metabolites and biomarkers in ageing and illness, metabolomics has gained popularity and has a wide range of applications. We will give a comprehensive summary of what is currently known about metabolomics in studies of ageing, with a focus on biomarkers for frailty. Metabolites related to amino acids, lipids, carbohydrates, and redox metabolism may function as biomarkers of ageing and/or frailty development, based on data obtained from human studies. However, there is a complexity that underpins biological ageing, due to both genetic and environmental factors that play a role in orchestrating the ageing process. Therefore, there is a critical need to identify pathways that contribute to functional decline in people with frailty.

## 1 Introduction

The introduction and application of -omics technologies, such as genomics, transcriptomics, proteomics, and metabolomics has revolutionised the study of ageing and human disease (Psychogios et al., 2011). Over the last 20 years, -omics discoveries have moved field of ageing research forward immensely, both in aiding better understanding the pathogenesis of age-related disease and in identifying potential biomarkers of advanced ageing and frailty. The discovery of the association between alleles of Alipo-protein E (APOE) and polymorphisms in Huntingtin (HTT) genes to the development of Alzheimer’s and Huntington’s disease respectively has changed our understanding of the inherited risk of age-related diseases (Artiga et al., 1998; Kegel et al., 2000). Serum biomarkers have now been identified for a variety of diseases associated with ageing including cardiovascular disease (Melander et al., 2015), and are able to indicate the presence of tumour-associated cells such as cancer-associated fibroblasts in breast cancer (Costa et al., 2018), and ovarian cancer (Givel et al., 2018). Circulating protein carbonyl groups have been proposed as biomarkers for oxidative stress in age-related diseases such as diabetes mellitus, rheumatoid arthritis, and Alzheimer’s disease (Dalle-Donne et al., 2003), and high levels of oxidative stress are associated with chronic inflammatory diseases. The application of metabolomics to identify age-related metabolites specific to frailty and healthy ageing is still in its infancy and this review will focus on our current understanding of the altered metabolome in ageing and frailty, and the potential for the development of clinical diagnostics based on metabolomics analysis.

## 2 What is metabolomics?

Metabolomics is the study and quantification of low-molecular weight metabolites, typically under 1.5 kDa (Zhang et al., 2012). Metabolomics maps the end products of a pathway (metabolites) including molecules such as amino acids, lipids, peptides, nucleic acids, and carbohydrates. Metabolites are indicators of the biological and pathological processes within a cell or the body. They can indicate the changes in response to factors such as diet, lifestyle, exercise, and clinical interventions (Zhang et al., 2012). Metabolomics tools have already been implemented with success in studies that investigated metabolism associated with inflammation, e.g., diabetes mellitus, heart failure, obesity, and cancer (Lokhov et al., 2016). More specifically, in cancerous cells, it was found that altered metabolism, and an increase in glucose uptake, facilitates rapid growth, compared to the control groups (Belfiore et al., 2009).

There are two distinct methods to perform metabolomics, targeted and untargeted. Targeted metabolomics investigates a known metabolite or set of metabolites in order to quantify them in a sample. This type of analysis is hypothesis-driven and provides absolute quantification of known metabolites within a biological sample. Untargeted metabolomics provides a profile of the entire metabolome and can be used to screen for new biomarkers of illness. It is often hypothesis-generating and provides relative quantification of global metabolite levels, including metabolites of unknown identity. It is often described as generating a “metabolic fingerprint” of a biological sample. Currently, no specific metabolites have been clinically linked to frailty, and therefore, untargeted metabolomics analysis is the most appropriate method for discovering biomarkers of early diagnosis, disease severity and therapeutic efficacy within frailty.

The main platforms for detecting metabolites currently in use are Nuclear Magnetic Resonance (NMR) spectroscopy, Liquid Chromatography-Mass Spectrometry (LC-MS) and Gas Chromatography-Mass Spectrometry (GC-MS) (Zhang et al., 2012). Mass spectrometry (MS)-based platforms are the most common methods for metabolomics (Wang et al., 2023). MS determines metabolites based on the mass-to-charge ratio of ions within the sample. Metabolites are separated based on their chemical properties using a specific choice of column, such as ion exchange, reverse phase, and hydrophobic interactions. These methods are highly robust and relatively sensitive which gives them the advantage to be used regularly and measure hundreds of metabolites (Zhang et al., 2012). ^1^H-NMR provides information based on the ^1^H atoms present in a molecule. One benefit of NMR metabolomics is that it is non-destructive, therefore, the sample can be processed and re-analysed if required. Unlike NMR, MS is destructive, and therefore, the samples are destroyed during analysis which makes re-analysis by MS impossible. Reference libraries such as Chenomx and HMDB can then be used to assign spectral NMR peaks in order to identify metabolites compared to standards (Joesten and Kennedy, 2019).

## 3 Metabolomics and frailty

### 3.1 How do we define frailty?

Life expectancy decreased for the first time ever from 2019 to 2021 in 28 different high-income countries (Schöley et al., 2022). Officially, COVID-19 deaths explained the deficit in life expectancy in the year 2021 across Europe and the United States, with the Netherlands being the only exception (Schöley et al., 2022). Despite this, the ageing population increases year upon year, with the global projected number of adults over the age of 65 increasing from 12% to 22% within the next 30 years (Organization, 2021). For the first time in history, there are more people aged over 60 years old compared to children under 5 years old (Organization, 2021)

The societal effects of an ageing population will have a greater impact in certain countries. For example, the current population over the age of 65 in Japan is already 28.7% as of 2021 (Aung et al., 2021). This is already having huge impacts on the health system and wider economy, as well as society as a whole. The resources needed to care for this population are increasing due to increasing demand (Hoogendijk et al., 2019). According to the 2022 consensus produced by the Royal College of Physicians, there is one full time geriatrician available per 8,031 people over the age of 65 across England (Waters, 2022). This is far from the British Society of Geriatricians target of one geriatrician per 500 people aged over 85 by the year 2030 (Gordon et al., 2023). The relatively new field of geroscience and the study of ageing is expanding year on year. It offers new perspectives on chronic age-related systemic inflammation and the associated conditions such as cardiovascular diseases, cancer, diabetes, and dementia (Kennedy et al., 2014).

Frailty is defined as the age-related loss of physiologic reserve and homeostatic capacity (Clegg et al., 2013). Frailty manifests as a multi-system decline, with a decrease in strength, energy, balance, immune function, cognition, mobility, eyesight and/or hearing (Fried et al., 2001). As a result, people who are frail are more susceptible to a range of adverse health-related events, such as falls, illness, disability, hospitalisation, institutionalisation, and mortality (Vetrano et al., 2019).

Frailty is strongly linked to socioeconomic status; lower education levels and low income are consistently associated with higher levels of frailty (Szanton et al., 2010; Franse et al., 2017; Hoogendijk et al., 2018). People from Asian and Black ethnic groups have an increased risk of becoming frail (Pradhananga et al., 2019) and notably, ethnic minority groups within majority white populations are at increased risk of developing frailty (Majid et al., 2020). The influence of gender on frailty is more complex; women have a higher risk of becoming frail. However, women also tend to live longer than men (Kane and Howlett, 2021). The intrinsic complexity of this syndrome and its pathology means there is no single diagnostic tool available to identify the degree of frailty. Some tools used to assess the severity of frailty are the frailty index and the frailty phenotype. Within these tools, there are multiple different types, such as the Fried frailty index (Fried et al., 2001), or the Rockwood clinical frailty scale (Rockwood et al., 2005). In England, all primary care systems have an electronic frailty index that scores all their older people aged 65 years and over into mild, moderate and severe frailty (Clegg et al., 2016).

Although frailty is associated with ageing, not all older people will develop frailty, and those that do have varying severities of frailty. Frailty has the potential to develop at a wide range of ages depending on the health status of the individual. More recently, frailty levels have reportedly increased among cohorts of middle-aged people from 1998 to 2018 (Blodgett et al., 2021). However, the assessment tool used in this study was not validated in people under the age of 70 (Searle et al., 2008). Currently, the tools used to assess frailty are validated in those over the age of at least 65. Frailty classifications are based on physical and cognitive assessments. New information obtained from correlating intrinsic biomarkers may complement current measures. They could allow the tracking of frailty progression, and support the clinical decisions made by healthcare professionals. This would make metabolomics a valuable tool for personalised medicine, and prevention and/or mitigation of disease.

The application of metabolomics to the study of frailty is new. To date, no metabolites, or biomarkers in general, have been specifically used to diagnose frailty syndrome. There have been studies that associate frailty with metabolic syndrome (Pérez-Tasigchana et al., 2017). There is also evidence that frailty is correlated with altered glucose metabolism (Kalyani et al., 2012). Therefore, metabolic biomarkers could be an interesting source of information regarding frailty development.

Frailty biomarkers are needed for predicting the degree of frailty on a cellular level, which would facilitate the early diagnosis of frailty, before significant physical symptoms manifest. Biomarkers would also aid in the prognosis of frailty within a clinical setting. There are longitudinal studies providing evidence that frailty is treatable, preventable, and reversible if detected early (Hoogendijk et al., 2019). Therefore, the identification of dysregulated metabolic pathways within frailty may provide new avenues for therapeutic targeting. It is likely that more than one biomarker will be needed for diagnostic accuracy. For this reason, potential biomarkers must be as precise as possible for clinicians to reliably use them in practice, and therefore, research is needed in this field.

### 3.2 The role of inflammageing in ageing and frailty

**FIGURE 1:**
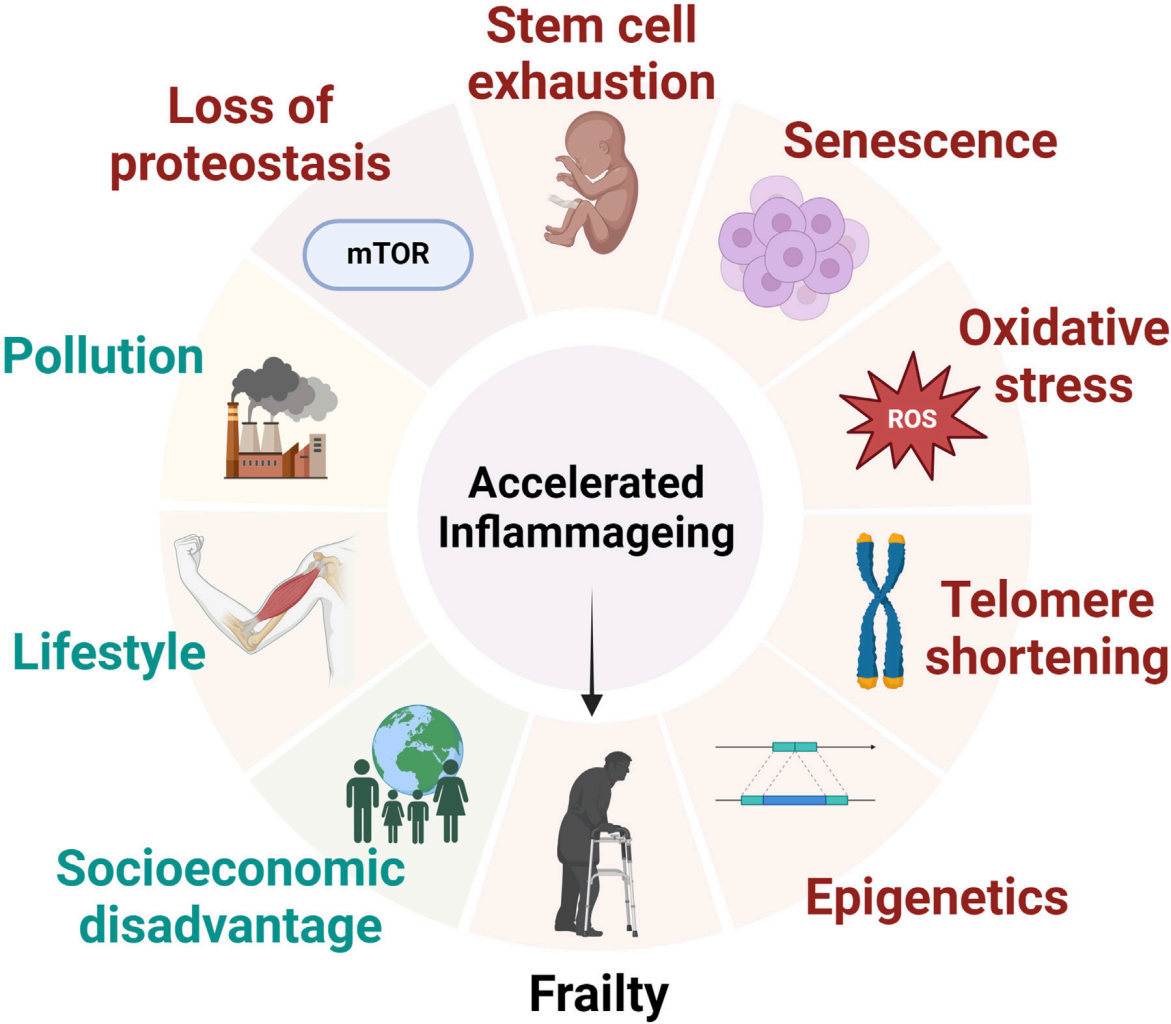
The major factors that contribute to the development of inflammageing and frailty status. Inflammageing is a consequence of environmental, cellular and genetic factors leading to alterations to intracellular homeostasis and ultimately contribution to the development of frailty syndrome.

There are seven “pillars” of ageing that are most reported in the wider literature. These include inflammageing, epigenetics, proteostasis, metabolic regulation, stem cell function, adaption to stress, and macromolecular damage (Figure 1) (Kennedy et al., 2014). The immune system plays a significant role in each of these pillars, and it has been proposed that controlling the immune response could have anti-ageing effects (Franceschi et al., 2018). Recent advances within the field of inflammageing field includes the establishment of “biological age” which can be calculated using metabolomics in combination with genomics. Biological age is distinct from chronological age, as biological age can be affected by lifestyle factors, genetics, and the environment (Jylhävä et al., 2017).

Changes to the immune system in older people are commonly associated with frailty; these include a decline in immune function leading to an increased risk of infections, and inflammageing which is defined as a state of low-grade chronic inflammation. Inflammageing is often associated with increased levels of circulating cytokines (e.g., TNFa, interleukin-6) as well as raised systemic markers of inflammation such as C-reactive protein (CRP) (Wilson et al., 2017). Inflammageing is caused by a number of factors such as the accumulation of senescent cells and oxidative stress, physical inactivity and obesity, lifestyle/environmental factors, e.g., visceral fat deposition, the decline of cellular processes such as mitochondrial function, autophagy and mitophagy, and genetic predisposition. This leads to an over-activation of pro-inflammatory pathways and an imbalance of immune function over time.

**FIGURE 2:**
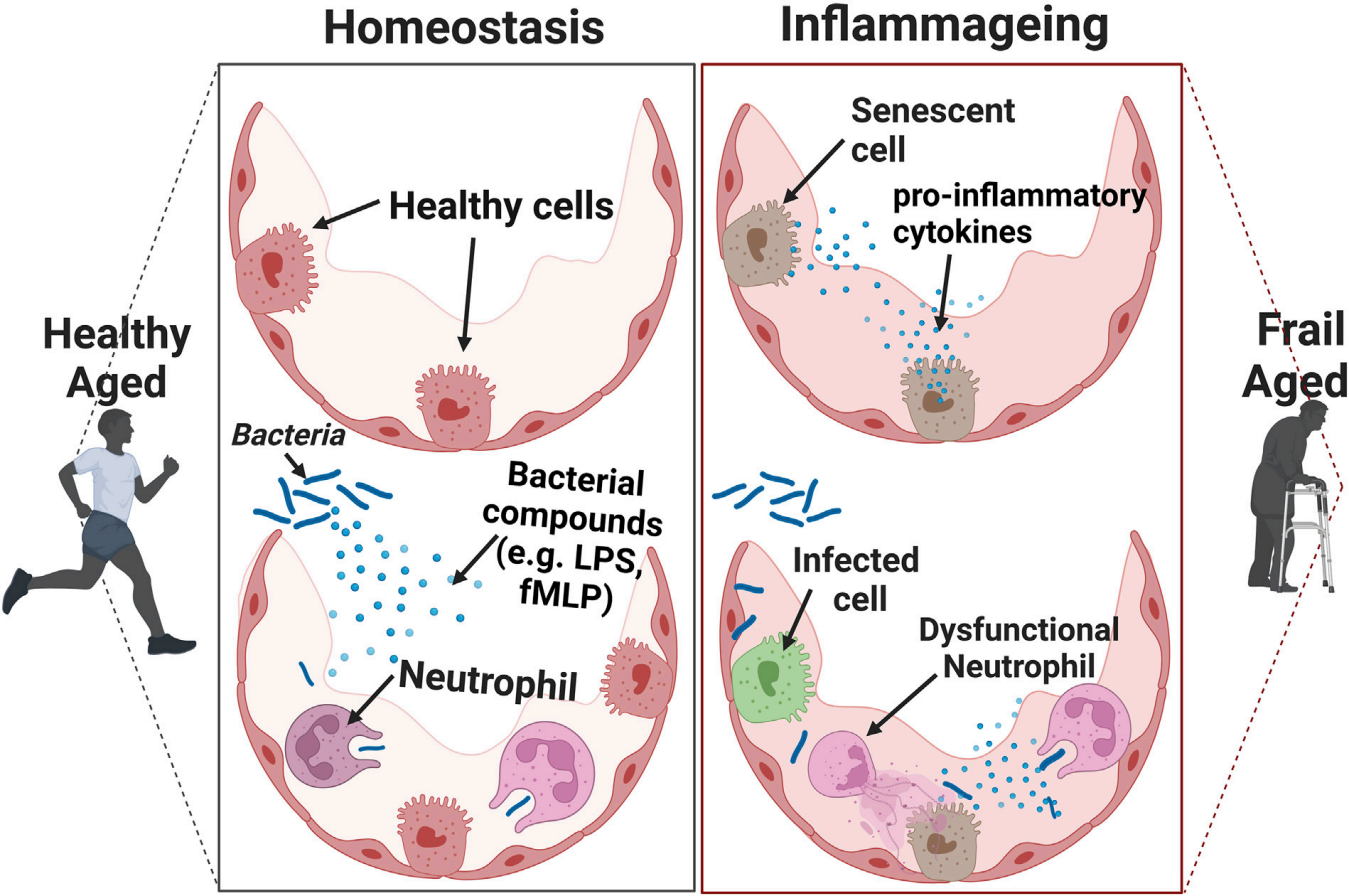
The effects of inflammageing on the immune response. Cells that have a senescent associated secretory phenotype (SASP) secret pro-inflammatory cytokines. This acts as a chemoattractant to induce immune-mediated clearance of senescent cells. When the number of senescent cells is relatively high in the presence of an infection, it can result in an inadequate clearance of pathogens.

Immune cell function declines with normal ageing (Hazeldine et al., 2014; Joesten and Kennedy, 2019). Several studies have identified a dysregulation of neutrophil function in older adults (Figure 2). For example, neutrophils from older people have a lower bacterial killing capacity (Wenisch et al., 2000) and they are less efficient at migration from the blood to sites of infection (Sapey et al., 2014). It is theorised that inaccurate neutrophil migration leads to neutrophils entering healthy tissues, then re-entering the vasculature thus puncturing the blood vessels unnecessarily. This is termed neutrophil reverse migration (Buckley et al., 2006) and can cause damage to the local blood vessels and tissues, promoting systemic inflammation in the process (Xu et al., 2022). There is also evidence from animal studies that normal neutrophil functions such as NETosis are impaired with older age (Xu et al., 2017). However, the alterations in neutrophil function associated with ageing is currently under-studied in humans and the current literature focuses largely on animal and *in vivo* studies (Xu et al., 2022). It is also established that with ageing there is an increase in neutrophil to lymphocyte ratio, and a decreased production of B-cells and T-cells (Nikolich-Žugich, 2018). This may explain why older people have an impaired response to both infections and vaccines (Goncalves-Mendes et al., 2019). The mechanisms for the decline in immune function with age, and whether this is irreversible, or could be therapeutically targeted, are not yet fully understood.

Being a relatively new field, there are huge gaps in the literature on how immune cell phenotype and function changes within inflammageing and healthy ageing. Defining these changes for each leukocyte may uncover the processes that can be targeted to mitigate inflammageing. For example, older people have higher mortality from COVID-19 (Polidori et al., 2021) and understanding metabolomics in this age group, including frailty, could potentially improve our understanding of this increased mortality and lead to interventions in the future. There is heterogeneity in the severity of COVID-19 among older people. Those who are frail and multi-morbid have a higher susceptibility to severe sepsis and death from COVID-19 (Polidori et al., 2021). Compared to healthy older adults receiving COVID-19 vaccines, nursing home residents showed higher vaccine-related fatality rate (Wyller et al., 2021). The potential vaccine-related fatal events may be linked to frailty, but not older age *per se* (Wyller et al., 2021). Another study found that the efficacy of COVID-19 vaccines against hospitalisation were consistently higher in people who were over 65 and classed as robust (65% efficacy) compared to people classed as frail (36% efficacy) (Tang et al., 2022). Therefore, the identification of frail individuals amongst the general population could identify those at highest risk virus-related mortality.

### 3.3 The contribution of oxidative stress to ageing and frailty

Increased oxidative stress is considered one of the hallmarks of ageing (López-Otín et al., 2013). Free radicals are reactive species that have unpaired electrons and can be charged or neutral. Some commonly known reactive species include superoxide anion, hydrogen peroxide, and hypochlorous acid which are collectively known as reactive oxygen species (ROS) since they contain oxygen. Other types of free radicals include nitric oxide which is a reactive nitrogen species (RNS) and hydrogen sulphide (reactive sulphur species). In order to produce energy, which is stored as adenosine triphosphate (ATP), mitochondria primarily produce reactive oxygen species (ROS). Superoxide anion is produced in the mitochondria and is the largest source of free radicals. In order to prevent free radicals from reacting with biomolecules, and causing damage that is irreparable, a number of molecular species, referred to as antioxidants, act to neutralise free radicals. “Oxidative stress” was precisely described as an imbalance favouring oxidants over antioxidants (Sies and Cadenas, 1985). High levels of oxidative stress as a result of mitochondrial dysfunction were one of the first pillars of ageing to be addressed (Harman, 1972). Although there are harmful consequences of oxidative stress, steady-state levels of reactive oxygen/nitrogen species (ROS/RNS) are required to activate transcription factors of particular genes that induce antioxidant defences. These pathways are also referred to as redox signalling.

Glutathione is an antioxidant synthesised within cells from three amino acids, cysteine, glutamine, and glycine. It has long been established that the production of glutathione decreases with ageing and within chronic disease states (Samiec et al., 1998; Lang et al., 2000; Uchmanowicz, 2020; Kumar et al., 2023). A recent randomised, placebo-controlled trial assessed the effects of supplementing glycine and N-acetylcysteine, the pre-cursors to glutathione, to young and older participants. Physical function was significantly improved in the older group receiving the treatment, including a faster walking speed over a shorter distance, and lowered systolic blood pressure, as well as lowered circulating IL-6 after 16 weeks. No changes were observed in the older person placebo group (Kumar et al., 2023).

The supplementation of various antioxidants including polyunsaturated fatty acids from fish oil and nicotinamide riboside (the pre-cursor to nicotinamide adenine dinucleotide, NAD) showed improved outcomes for people aged over 60, including improved mitochondrial antioxidant capacity, preserved muscle mass, increased strength, and quality of life (Bo et al., 2019; Lippi et al., 2022a). These improvements were also observed after exercise interventions in elderly individuals, in various randomised controlled trials (Lippi et al., 2022b).

Ergothioneine is an antioxidant and ROS scavenger found in red blood cells (Chen et al., 2024). Decreased levels of S-methyl-ergothioneine, a metabolite of ergothioneine, has been reported in people over the age of 65 with frailty, Alzheimer’s disease and dementia (Kameda et al., 2020; Kondoh et al., 2022). Ubiquinone, or coenzyme Q10 (CoQ10), is a key element in decreasing the oxidative damage caused to lipoproteins and maintaining antioxidant capacity within mitochondria. CoQ10 was found to be significantly decreased in the cerebrospinal fluid in people with multiple system atrophy and Parkinson’s disease (Du et al., 2018). Higher CoQ10 levels in plasma were associated with a significantly lower risk of cardiovascular disease, measured by the quotient total cholesterol/HDL cholesterol, in people over the age of 65 (de la Bella-Garzón et al., 2022). They were also found to be higher in women compared to men over the age of 65 (de la Bella-Garzón et al., 2022).

Cortisol is a glucocorticoid steroid hormone, produced in response to stressful environmental situations and mental stress. Cortisol drives the physical manifestation of stress, including work-related burnout, trauma, emotional distress, and environmental disturbances (Dapprich et al., 2021). It has been found to act on protein metabolism, inhibiting muscle synthesis, and activating gluconeogenesis (Athanasouli et al., 2021). Circulating cortisol levels have been found to increase with age (Yanagita et al., 2020). One study found that cortisol levels were increased with frailty severity, however, there were no significant biomarkers related to oxidative stress identified (Marcos-Pérez et al., 2019). This study utilised targeted metabolomics to measure reactive oxygen and nitrogen species levels in the serum of people aged 65–102 years (Marcos-Pérez et al., 2019).

The chronic inflammation observed in older people with frailty could be due to the compounded effects of diet and lifestyle throughout a lifetime. Many plant-derived compounds such as polyphenols and flavonoids have the capability to activate the redox signalling pathways such as NF-kB that also induce cytoprotective and antioxidant genes (Calabrese and Kozumbo, 2021). Tocopherol and tocotrienol are analogues of vitamin-E. They are well documented antioxidants due to their scavenging abilities for lipid peroxidation-derived free radicals and reactive nitrogen species (Ahsan et al., 2014). Additionally, carotenoids have antioxidant qualities that could prevent lipid peroxidation. The cleavage of β-carotene can yield all-trans-retinal (Grune et al., 2010), a polyene that binds to opsin to form rhodopsin, and acts as a key metabolite in vision (Rando, 1996). The NeuroExercise study at the German Sports University in Cologne, Germany, showed significant differences in plasma levels of six carotenoids and two tocopherols in subjects with mild-cognitive impairment (Gerger et al., 2019). Physical performance was also measured, and β-cryptoxanthin was associated with the timed-up and go test, and γ-tocopherol with the number of daily steps (Gerger et al., 2019).

Significant reductions in circulating vitamin-E have been reported amongst people with frailty (Erusalimsky et al., 2016) as well as lower circulating levels of carotenoids (Rietman et al., 2019). Along with the increased levels of pro-inflammatory cytokines such as interleukin-6 (IL-6), tumour necrosis factor alpha (TNFa), and C-reactive protein (CRP) reported in frailty, this indicates an imbalance in the pathways regulating inflammation, with an increase in oxidative stress in frailty. One study that focused solely on older women did not find any association between circulating vitamin-E levels and frailty. However, there was a significant association between total carotenoids and total micronutrient deficiency with frailty score (Semba et al., 2006). More recently, there was a significant association found between higher serum levels of IL-6 and lower β-cryptoxanthin and β-carotene between frail and non-frail cohorts. However, no differences were observed between serum levels of vitamin E or vitamin D (Kochlik et al., 2023). It is crucial to keep in mind that many studies looking for a link between vitamin E and frailty only measured the alpha-tocopherol form, despite the fact that it has been widely documented how various forms of both the tocopherol and tocotrienol families play a role in age-related cognitive decline (Mangialasche et al., 2012).

Vitamin D has a wide array of functions within humans, from regulating calcium homeostasis and mineralisation of bone tissue to differentiation and proliferation in cell types including cardiovascular, renal, and immune cells (Cardus et al., 2009). Vitamin D deficiency was associated with increased mortality in COVID-19 patients. People with severe vitamin D deficiency had a 50% mortality rate 10 days post-hospitalisation (Carpagnano et al., 2021). The production of vitamin D within the skin declines with age, and older nursing home patients who do not spend time outdoors are particularly at high risk (van Schoor and Lips, 2018). Increased bone fractures, muscle weakness, and falls have all been associated with vitamin D deficiency, all of which are more common amongst people with frailty (Bischoff-Ferrari et al., 2009; Lips et al., 2010). 25-hydroxyvitamin D (25(OH)D) within serum is a marker of circulating vitamin D levels. Frailty severity was significantly associated with lower circulating 25(OH)D (Marcos-Pérez et al., 2020). A recent intervention trial of vitamin D supplementation over a total of 24 months did not reduce the risk of falls or overall frailty score (Cai Y. et al., 2022). However, vitamin D supplementation did increase survival against COVID-19 infection in a study conducted on a frailty unit in France (Annweiler et al., 2020). The mortality rate after 14 days of COVID-19 infection for frail individuals receiving vitamin D supplementation was only 6.9%, compared to those who did not receive supplementation, in which the mortality rate was 31.3% (Annweiler et al., 2020). A study conducted in the U.K. showed no significant differences in mortality in elderly individuals with COVID-19, however, there were significant differences in the outcomes between people who had higher vitamin-D serum concentrations, compared to those who had lower circulating vitamin-D. The group with lower vitamin-D levels had higher circulating cytokines, CRP, and lactate dehydrogenase (LDH), and were more likely to become hypoxic and require ventilator support (Baktash et al., 2021).

Assessing antioxidant and cortisol profiles at the metabolic level could provide an insight into those who are at risk of becoming frail, and/or to assess the efficacy of clinical interventions before the physical manifestations emerge. Extensive research has proven the numerous health-promoting properties of antioxidants. However, evidence of their ability to reverse or prevent frailty is still lacking. Future studies and interventions on these compounds in relation to frailty would advance the knowledge in this emerging field.

### 3.4 Serum metabolites as markers of frailty

Sarcopenia is the loss of muscle mass and strength, along with an increased risk of physical disability, poor quality of life, and increased risk of death (Cruz-Jentoft et al., 2010). It is also one of the main factors contributing to frailty (Landi et al., 2015). Due to the protein catabolism that is observed within frailty and sarcopenia, utilising amino acid metabolites as biomarkers of frailty could prove useful. Age-related changes in amino acid levels have also been noted with ageing but with varying trends. It is more challenging to summarise the directions of amino acid changes within frailty. It has long been established that protein intake and resistance training has been associated with reduced frailty risk as well as an intervention for improving physical strength (Tieland et al., 2012). The branched chain amino acids (BCAAs) regulate the pathways involved in muscle synthesis (Volpi et al., 2003). These amino acids, specifically leucine, isoleucine, and valine have been shown to be lower in people with frailty. A lower concentration of systemic BCAAs was associated with sarcopenia and osteoporosis in older adults (Saeki et al., 2020). In a study of non-fasted plasma samples, there was a significant decrease of leucine and isoleucine between older adults with sarcopenia and the non-sarcopenic control group (Ottestad et al., 2018). In a 10-week intervention trial with leucine supplementation, a 25.4% increase in muscle strength and 3.6% increase in muscle volume was observed in older Japanese women with sarcopenia (Kim et al., 2012). More recent studies have additionally shown an association between daily leucine intake and muscle mass and strength in both males and females with frailty and those who were healthy (Lixandrão et al., 2021; Vega-Cabello et al., 2023). Although protein metabolism was not measured, it was probable that the increases in muscle strength observed were due to increased leucine-mediated protein synthesis. Conversely, in the Baltimore Longitudinal Study on Ageing (BLSA) found that older people with poor muscle quality had higher plasma levels of leucine, isoleucine, and methionine (Moaddel et al., 2016). However, this study included adults aged 50 and above, therefore, this difference could be due to the wider age-range included in the study.

In the BIOmarkers associated with Sarcopenia and PHysical frailty in EldeRly pErsons (BIOSPHERE) study, a model used a combination of nine amino acids to correctly predict frailty in up to 75% of cases (Calvani et al., 2018). These amino acids included asparagine, aspartic acid, citrulline, ethanolamine, glutamic acid, sarcosine, taurine, aminobutyric acid and methionine. A secondary analysis by the same group coupled their analysis with partial least squares–discriminant analysis (PLS-DA), and showed citrulline, asparagine, and aspartic acid as significantly higher in people with frailty and sarcopenia (Calvani et al., 2021). However, other studies have shown no differences in the levels of asparagine and aspartic acid amongst severely frail and robust individuals (Adachi et al., 2018). Interestingly, citrulline was found to be lower in centenarians (Cai D. et al., 2022). One study looking at frailty and sarcopenia, as two different disease states, identified 15 biomarkers distinctly associated with frailty and 22 associated with sarcopenia (Kameda et al., 2021). Of the 22 biomarkers found, only one of them, aspartate, was significantly increased within sarcopenia. However, there have been studies in which aspartate is also increased within frailty (Adachi et al., 2018; Calvani et al., 2021). High levels of 3-methyl-histidine have been reported as an indicator for physical frailty, as it is involved in muscle catabolism (Moaddel et al., 2024).

One of the well-established metabolite groups associated with ageing and frailty are lipids. It is estimated that there are around 1200 fatty acids in humans (Wishart et al., 2012). Due to the vast assortment in molecular structure and the biochemical pathways associated with lipids, there can be an uncertainty as to where the significant metabolites originate from. As a result, most studies on frailty have focused largely on amino acids and omitted the role of lipid metabolites in frailty (Saedi et al., 2019). Carnitines, acyl-carnitines, and acetyl-carnitines regulate fatty-acid oxidation, as well as protecting the cellular membrane and preventing accumulation of fatty acids, and their key roles have recently been reviewed (Virmani and Cirulli, 2022). Several studies have displayed an association between physical performance, such as weakness, slowness, and fatigue with higher levels of circulating fatty acids such as palmitoylcarnitine and stearoylcarnitine (Lum et al., 2011; Pujos-Guillot et al., 2019; Pan et al., 2022). However, there were significant differences found between men and women (Pujos-Guillot et al., 2019) which is thought to be due to the increase in circulating lipids in women after menopause (Auro et al., 2014). Similarly in patients receiving dialysis treatment, there was a significant correlation between reduced physical function and acylcarnitine species detected in plasma (Murphy et al., 2012), and in elderly women with breast cancer (Corona et al., 2014). On the contrary, there have been studies that show a decrease in the levels of branched chain amino acids and acylcarnitines in older people with frailty as well as healthy older people (Fazelzadeh et al., 2016) as their levels tend to decrease with age.

It is reported that the metabolism of phospholipids changes with ageing and in frailty, however, there are varying reports on the specific phospholipid molecules that are changed (Yu et al., 2012; Collino et al., 2013; Pradas et al., 2019). This is likely due to the regional and ethnic differences between studies, as well as the culture and lifestyle habits between people and countries.

### 3.5 Sex differences and metabolism

It is now well established that oxidative stress plays a key role in the onset of inflammageing. However, significant differences have been reported between males and females and the level of antioxidant metabolites with ageing. First, pro-oxidant enzymes including xanthine oxidase (XO) and NADPH-oxidase (NOX) are more active in males (de Toda et al., 2023). Additionally, males exhibit decreased levels of superoxide dismutase (SOD) and glutathione peroxidase expression and activity, two crucial antioxidant enzymes (de Toda et al., 2023). This could be one of the contributing factors for longer lifespan seen within females compared to males. Malondialdehyde (MDA) is a product of lipid peroxidation and is highly toxic, and due to this it is a reliable marker for oxidative stress (Giera et al., 2012). Massudi et al. (Massudi et al., 2012) found a significant positive correlation between MDA formation and age within men but not women. However, they also found significant increases in phosphorylated-H2AX, a marker for DNA damage, in both males and females with age.

Clinical data shows that although women live longer than men, they have a higher prevalence of frailty in older age (Kane and Howlett, 2021). The Karlsruhe Metabolomics and Nutrition (KarMeN) study in Germany found that menopause status was predicted with up to 88% accuracy based on the metabolic profile in participants up to the age of 80 (Rist et al., 2017). It is thought that the differences in sex hormones modulate the cellular composition and function of immune cells, and therefore, contributes to the differences in lifespan and frailty onset (Gubbels Bupp et al., 2018). One study found that cholesterol and triglyceride levels increased around the age of 30 in men but didn’t increase in women until the age of 50 (Auro et al., 2014). The authors stated this difference could be driven by menopause. One study did not find any significant differences between metabolites based on race or sex among black and white males and females but did find differences associated with frailty score (Marron et al., 2024).

### 3.6 Metabolic changes associated with cognitive frailty

Mild cognitive impairment (MCI) is reported as memory loss with a quantifiable cognitive deficit, but is not severe enough to affect daily activities (Jackson et al., 2016). Cognitive frailty is defined as the presence of MCI in people with physical frailty (Kelaiditi et al., 2013). It can progress into dementia if the acquired cognitive impairments become severe (Hugo and Ganguli, 2014). It is predicted that the growth rate from cognitive frailty to dementia development is between 5%–15% annually (Liu et al., 2018).

The dysregulation of kynurenine, a metabolite of tryptophan, is involved in neuromuscular dysfunction (Westbrook et al., 2020). Previous studies have shown an association between higher levels of circulating kynurenine and Alzhemier’s disease (Baran et al., 1999), depression (Myint et al., 2012; Dantzer, 2017) and stroke (Darlington et al., 2007). Increased circulating kynurenine has been observed in people with frailty and was associated with circulating TNF-α and IL-6 levels, a slower walking speed and weaker grip strength (Marcos-Pérez et al., 2017; Westbrook et al., 2020). One study showed a 34.3% increase of serum kynurenine in people with frailty compared to those considered pre-frail (Jang et al., 2020). A recent study identified kynurenine in combination with cysteine, glutamine, citrulline, tyrosine, and phenylalanine as potential diagnostic biomarkers for frailty (Zhou et al., 2024). Additionally, many studies have suggested that kynurenine induces cytotoxicity in other cells and tissues such as neuronal and chronic pain (Jovanovic et al., 2020), renal (Addi et al., 2018), major depressive disorder in females (Zhou et al., 2019) and retinal disease (Rejdak et al., 2011). Due to this, both kynurenine and its associated down-stream metabolites could explain the multi-system decline observed in people with frailty. Due to the occurrence of cognitive decline simultaneously with frailty, there is a lot of overlap of metabolite markers such as 3-methyl-histidine, methionine, tryptophan, and S-methyl-ergothioneine (Teruya et al., 2021).

Interestingly, four metabolites found to be increased in robust elderly people compared to people with dementia were caffeine and derivatives of caffeine (Teruya et al., 2021). There is increasing evidence that demonstrate the benefits of acute caffeine and matcha consumption, which lead to improvements in cognitive function (Sohail et al., 2021) as well as physical function in endurance (Southward et al., 2018), and in muscular strength (Grgic et al., 2020). This could be due to the ferulic chlorogenic, caffeic, and n-coumaric acids contained in in caffeine which provide antioxidant capacity (Farah and Donangelo, 2006).

A recent study followed the signature of blood metabolites in amyloid-positive older people with a mean age of 77 over 4 years. 9 metabolites including methionine, glucose, serine, citrate, hydroxybuterate, succinate, acetone, sphingomyelin d18:1/C26:0 and triglyceride C48:3 were reported as predictive markers up to 3 years before the onset of cognitive decline (Tremblay-Franco et al., 2024).

## 4 Insights into metabolism and ageing from centenarians

**FIGURE 3:**
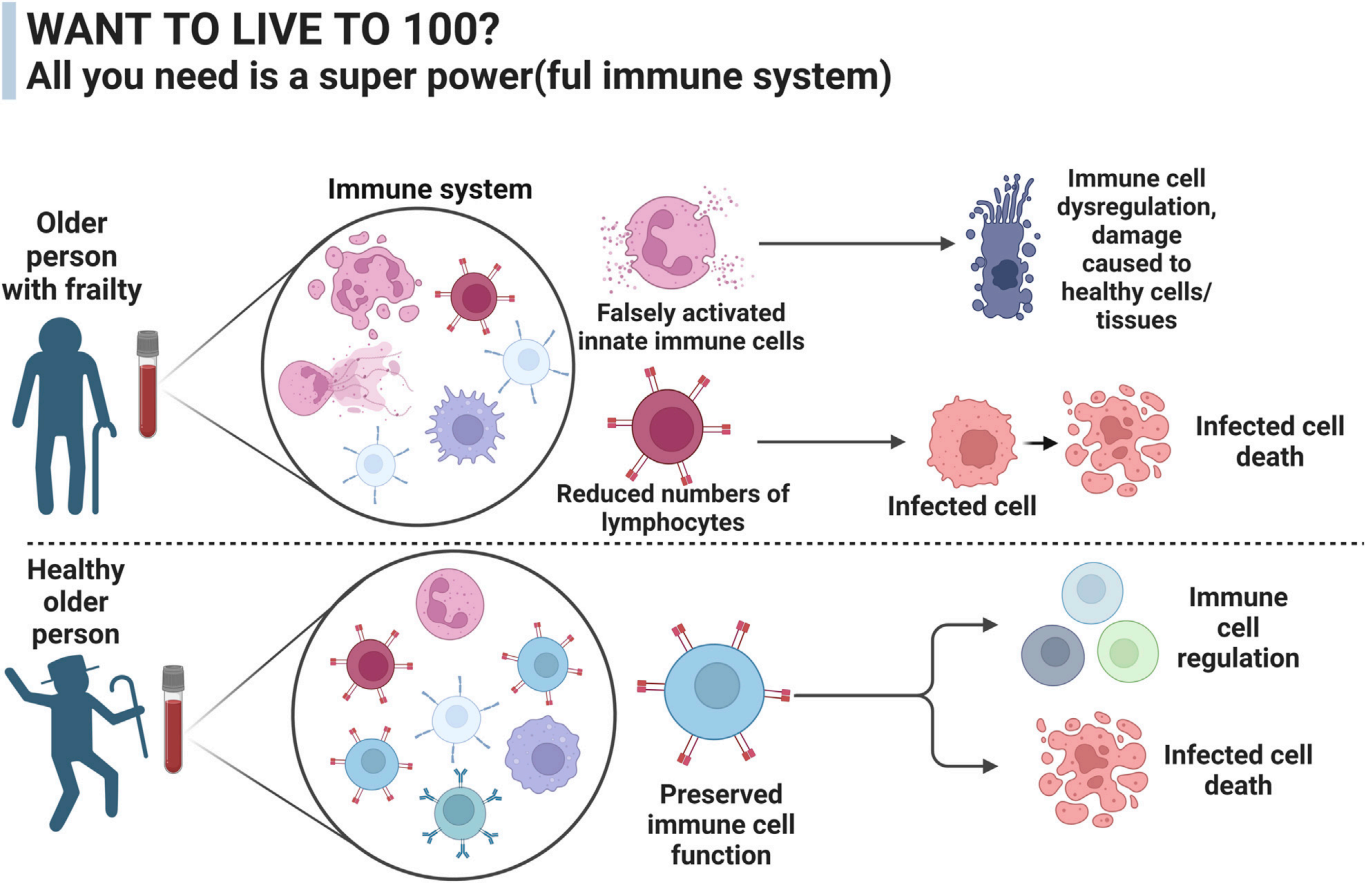
The difference in immune function between a healthy aged person and a person with frailty syndrome. The immune system plays an important role in how people age and can alter the development of debilitating diseases such as cancer and sarcopenia, two major contributing factors to frailty development.

Centenarians are classified as people who survive until the age of 100. The global number of centenarians is predicted to quintuple by 2050 (Willcox et al., 2010). In 2015, DNA methylation levels of peripheral blood mononuclear cells (PBMCs) showed that centenarians are 8.6 years younger than expected from their chronological age (Perls, 2004). In order to maintain and restore equilibrium in complex biological systems, we need to be able to recognise and quantify the characteristics of resilience. These processes might include greater stress management, increased energy efficiency, or improved immunity, as demonstrated in centenarians (Figure 3). Centenarians are living longer than people of the same birth cohorts and are regarded as the prime example of healthy ageing.

The incidence of age-related chronic diseases among centenarians is lower compared to other older people (Evert et al., 2003; Pavlidis et al., 2012; Borras et al., 2020; Cruces-Salguero et al., 2023). Compared to other older people, centenarians have lower levels of circulating triglycerides, cholesterol, and glucose (Camacho-Pereira et al., 2016; Murata et al., 2024). This reflects a better cardiovascular risk profile with preserved insulin sensitivity due to lower glucose levels (Camacho-Pereira et al., 2016). The reduced form of nicotinamide adenine dinucleotide (NADH) has been reported in lower concentrations with ageing and within sarcopenia, across different ethnicities (Massudi et al., 2012; Migliavacca et al., 2019; Sanchez-Roman et al., 2022). In centenarians, NAD^+^ and NADH were positively correlated with cognitive function, and no differences were found between centenarians and the younger healthy controls (Wadhera et al., 2016). Low density lipoproteins (LDL) are positively correlated with cardiovascular disease morbidity (Aiello et al., 2021). LDL levels were reported higher in adults aged 40–89 compared to centenarians in Sicily (Fugger et al., 2020). No significant differences were found between the centenarians and younger people aged 18–39 years old.

## 5 Accelerated ageing in chronic disease

Certain conditions, regardless of age, can accelerate the ageing process and shorten lifespan. Autoimmune diseases are associated with altered metabolism, early-onset immunosenescence, and poor clinical outcomes (Wu et al., 2007). The dysregulated metabolic signalling within autoimmune diseases contributes to increased oxidative stress, which compromises cellular integrity. Regardless of age, telomere length was found to be shortened in neutrophils in people with systemic lupus erythematosus (SLE) (Blinova et al., 2016), and in mononuclear cells in people with rheumatoid arthritis (Krysko et al., 2019). Shorter leukocyte telomere length was associated with disease severity in people with multiple sclerosis (Levett et al., 2016).

People with chronic conditions may develop frailty prematurely. Previous studies have reported that in individuals with human immunodeficiency virus (HIV) infection, 5%–29% developed frailty (Afilalo, 2011) as well as 25%–50% of patients with cardiovascular disease, depending on the population and frailty scale used (Haider et al., 2019). Rheumatoid arthritis (RA) is an autoimmune disease that results in systemic inflammation. The presence of frailty in people with RA is higher than the national average, with reports ranging from 15%–39% in RA cohorts under the age of 65, compared to 12% in the general population over 65 (Tada et al., 2019; Minamino et al., 2021). It is important to note that the frailty indexes used in these reports were not validated in people under 65; there is no current frailty assessment tool that has been designed to diagnose frailty in younger people. Therefore, the index validated in people over 65 may not be accurately applicable to those under 65. Although the participants were not over 65, they qualified as frail according to these measurement parameters. This is one example of accelerated frailty development within chronic inflammatory disease. Similarly to older people with frailty, reduced dietary protein and fish intake, along with malnutrition, is implicated in frailty development in people with RA (Urbaniak et al., 2019; Minamino et al., 2021; Tański et al., 2021).

As seen within people with frailty, the amino acids valine, leucine, isoleucine, phenylalanine, and tryptophan are consistently reported to be lower in people with RA (Ouyang et al., 2011; Zhou et al., 2016), SLE (Herman et al., 2019), and multiple sclerosis (Yang et al., 2015). This suggests increased muscle degradation into amino acids in response to high energy demands, and increased inflammatory responses associated with each disease. Valine in particular acts as a donor substrate to the tricarboxylic acid (TCA) cycle, and long-term energy demands may lead to the erosion of valine stores (Mesinovic et al., 2019). Accelerated muscle atrophy has been reported in those with type 2 diabetes (Meex et al., 2019), and this is associated with lipo-toxicity (Wittenbecher et al., 2022). As shown in frailty, isoleucine, leucine, valine, alanine, and short-chain acyl-carnitines levels were associated with type-2-diabetes risk in a 10-year follow up study in nurses (Bestilny et al., 2000).

People with HIV developed an acceleration of age-related methylation changes of 13.7–14.7 years compared to HIV-negative individuals (Rickabaugh et al., 2015). This is thought to be due to the considerable stress placed on the immune system, and shortened telomere length associated with T-cells in the HIV disease state (Breen et al., 2022). In a recent longitudinal study, it was found that telomere length of PBMCs decreased significantly in individuals with HIV, during a 3-year period, compared to age-matched controls (Starup-Linde et al., 2020). People with HIV also present with higher prevalence of osteoporosis and lower bone-mineral density, similarly to that observed with elderly age (Frias et al., 2024). As seen in frailty, kynurenine was recently reported higher in people living with HIV compared to controls (Cassol et al., 2014). The kynurenine to tryptophan ratio, myoinositol, glutamate, N-acetylaspartate, and beta-hydroxybutyric acid correlated with worse cognitive test scores in people with HIV (Zhang et al., 2022) similarly to those seen in people with neurodegeneration and cognitive frailty (Westbrook et al., 2020; Al-Aly et al., 2021).

Beyond the initial acute SARS-CoV-2 virus infection (COVID-19), people who recover from COVID-19 may endure post-acute sequelae, also known colloquially as long-Covid (Al-Aly et al., 2021). Long-Covid is associated with chronic kidney problems, higher risk of ischemic stroke and joint pain (Al-Aly et al., 2021; Xu et al., 2022). Increased kynurenine and decreased tryptophan were found in people with critical COVID-19 infections (Danlos et al., 2021). A by-product of tryptophan metabolism, indole-3-acetic acid, discriminated between COVID-19 positive and negative patients. The kynurenine metabolite, anthranilic acid, also predicted poor prognosis (Danlos et al., 2021). Between 3 and 10 months after COVID-19 infection, plasma levels of triglycerides, cholesterol, and phospholipids were increased (Bizkarguenaga et al., 2022). Interestingly, most of the participants in this study were reportedly asymptomatic or had mild COVID-19 symptoms. The raised lipid levels reported could be contributing to the increased risk of atherosclerosis observed following COVID-19 infection (Liu and Zhang, 2021).

## 6 Summary and final conclusion

In this review we have highlighted the important role that metabolism plays in the pathophysiology of frailty and ageing. We have summarised the effects of inflammageing and oxidative stress on biological ageing, and identified a key role of antioxidant, lipid, vitamin and amino acid metabolites on frailty. Whilst it is likely that a set of metabolites, rather than one, will be identified as a diagnostic for frailty, it is possible that a single metabolite may eventually be identified which can be used together with other genetic or clinical biomarkers to identify older people at risk of developing frailty. One study used machine learning models to predict chronological age based on haematological data, including metabolites such as glucose, cholesterol, and triglycerides (Mamoshina et al., 2018). The predictions were more accurate by training these models on combined datasets, or population-specific data. Ideally, metabolites categorised by tissue or cell types would be more specific, due to the metabolic variation between systems. Additionally, understanding the mechanisms of ageing requires the quantification of metabolic changes across the human life cycle. However, longitudinal metabolomics data are uncommon, and such studies would require careful experimental design to minimise the effect of ethnicity, lifestyle, and diet on metabolism. We have outlined how metabolomics has been used to monitor disease progression, including how individuals respond to therapeutic interventions. Disturbances to the metabolome indicates one or more metabolic pathways that have been dysregulated, and therefore, can be used as a precise indicator compared to standard biomarkers. Additionally, the metabolome could aid in identifying new therapeutic targets in human diseases, by providing insight into the whole physiology of an individual.
